# Oomycete Gene Table: an online database for comparative genomic analyses of the oomycete microorganisms

**DOI:** 10.1093/database/baz082

**Published:** 2019-06-29

**Authors:** Thidarat Rujirawat, Preecha Patumcharoenpol, Weerayuth Kittichotirat, Theerapong Krajaejun

**Affiliations:** 1 Department of Pathology, Faculty of Medicine, Ramathibodi Hospital, Mahidol University, Rama 6 Road, Ratchathewi District, Bangkok 10400, Thailand; 2 Research Center, Faculty of Medicine, Ramathibodi Hospital, Mahidol University, Rama 6 Road, Ratchathewi District, Bangkok 10400, Thailand; 3 Molecular Medicine Program, Multidisciplinary Unit, Faculty of Science, Mahidol University, Rama 6 Road, Ratchathewi District, Bangkok 10400, Thailand; 4 Interdisciplinary Graduate Program in Bioscience, Faculty of Science, Kasetsart University, Ngamwongwan Road, Jatujak District, Bangkok 10900, Thailand; 5 Systems Biology and Bioinformatics Research Group, Pilot Plant Development and Training Institute, King Mongkut’s University of Technology Thonburi, Bang Khun Thian Chai Thale Road, Bang Khun Thian District, Bangkok 10150, Thailand; 6 Bioinformatics and Systems Biology Program, School of Bioresources and Technology and School of Information Technology, King Mongkut's University of Technology Thonburi, Bang Khun Thian Chai Thale Road, Bang Khun Thian District Bangkok 10150, Thailand

## Abstract

Oomycetes form a unique group of the fungal-like, aquatic, eukaryotic microorganisms. Lifestyle and pathogenicity of the oomycetes are diverse. Many pathogenic oomycetes affect a broad range of plants and cause enormous economic loss annually. Some pathogenic oomycetes cause destructive and deadly diseases in a variety of animals, including humans. No effective antimicrobial agent against the oomycetes is available. Genomic data of many oomycetes are currently available. Comparative analyses of the oomycete genomes must be performed to better understand the oomycete biology and virulence, as well as to identify conserved and biologically important proteins that are potential diagnostic and therapeutic targets of these organisms. However, a tool that facilitates comparative genomic studies of the oomycetes is lacking. Here, we described in detail the Oomycete Gene Table, which is an online user-friendly bioinformatic tool, designed to search, analyze, compare and visualize gene contents of 20 oomycetes in a customizable table. Genomic contents of other oomycete species, when available, can be added to the existing database. Some of the applications of the Oomycete Gene Table include investigations of phylogenomic relationships, as well as identifications of biologically important and pathogenesis-related genes of oomycetes. In summary, the Oomycete Gene Table is a simple and useful tool for comparative genomic analyses of oomycetes.

## Introduction

Oomycetes are fungal-like, aquatic, eukaryotic microorganisms that belong to the stramenopiles–alveolates–Rhizaria supergroup (1). Oomycetes include the organisms of the genera *Albugo*, *Aphanomyces*, *Bremia*, *Hyaloperonospora*, *Lagenidium*, *Peronospora*, *Phytophthora*, *Phytopythium, Plasmopara*, *Pythium* and *Saprolegnia* (2, 3). Lifestyle and pathogenicity of the oomycetes are diverse. Many pathogenic oomycetes (e.g. *Phytophthora* and *Pythium* species) affect a broad range of plants and cause enormous economic loss each year (4). Some pathogenic oomycetes (e.g. *Pythium insidiosum*, *Pythium aphanidermatum*, *Aphanomyces invadans*, *Saprolegnia parasitica* and *Lagenidium* species) infect a variety of animals, including humans, and can lead to a deadly disease (5–10). No effective antimicrobial agent against oomycetes is available, making the control of infections caused by these organisms difficult and challenging.

Genomes of many oomycetes have been sequenced and deposited in public repositories, such as the National Center for Biotechnology Information (NCBI), the DNA Data Bank of Japan and the European Bioinformatics Institute (6, 11–22). Availability of the genome data provides opportunities to explore biology, pathogenicity and evolution of oomycetes, which can lead to the discovery of a novel method for efficient control of the infections caused by these organisms. Comparative analyses of oomycete genomes must be performed to better understand the oomycete biology and virulence, as well as to identify conserved and biologically important proteins that are potential diagnostic and therapeutic targets of these organisms. An array of bioinformatic tools and well-trained personals are usually required for such complicated analyses. A few web-based tools, such as FungiDB (23) and EuMicrobeDBLite (24), have been developed for genomic analysis of oomycetes. These online tools provide bioinformatic features, which include sequence retrieval, basic local alignment search tool (BLAST) search engine, gene annotation, gene location and gene and protein architecture. However, a bioinformatic tool that facilitates the comparative genomic studies of oomycetes is still lacking.

Recently, we have developed an online user-friendly bioinformatic tool, called ‘Oomycete Gene Table’, to facilitate the comparative genomic analysis of the human pathogen *P. insidiosum* and 19 other oomycetes, as well as 2 diatoms (3). The Oomycete Gene Table can be used to search, retrieve, sort, filter, compare and display gene contents of the oomycete genome(s) of interest in a customizable and easy-to-understand table. The Oomycete Gene Table has been successfully used to investigate the phylogenomic relationships and identify the conserve and species-specific genes of *P. insidiosum* (3, 25). As only a few bioinformatic tools for oomycetes are available, the Oomycete Gene Table is an additional, simple and useful tool that can be applied for the genomic analyses of oomycetes. The key features of the Oomycete Gene Table are described in detail here to facilitate its utilization for genomic analyses of oomycetes. The Oomycete Gene Table is available online at http://www.sbi.kmutt.ac.th/cgi-bin/gt/viewer?organism=oomycetes&build=190208.

## Contents of the Oomycete Gene Table

**Figure 1:**
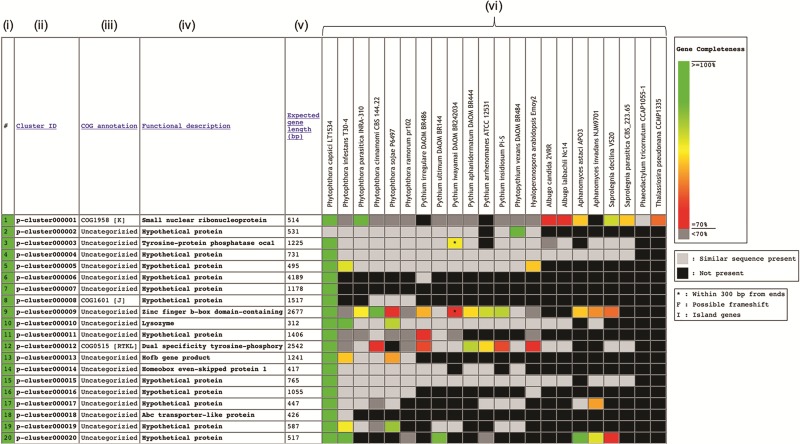
Components of the Oomycete Gene Table. Each row represents a gene cluster. Several columns are arranged to present **(i)** Row numbers, **(ii)** Cluster identification numbers, **(iii)** COG, **(iv)** Functional descriptions, **(v)** Expected gene lengths and **(vi)** organism names (e.g. 20 oomycetes and 2 diatoms). Percent completeness of a gene in each cell is indicated by color shading. A light-gray cell shows that a sequence with significant similarity (but without a complete open reading frame) is identified in a particular organism. A black cell indicates that no gene is identified in the corresponding genome.

The Oomycete Gene Table was constructed based on sequence similarity analyses of gene contents from 20 oomycetes and 2 diatoms, using an algorithm described by Kittichotirat *et al.* (26). Briefly, genes were clustered based on four different BLAST strategies using *E*-value of ≤10^−6^, pairwise sequence identity of ≥30% and pairwise sequence alignment coverage of ≥50% (3). This clustering process was iterated until the gene members of all clusters are stable or no longer change. The Oomycete Gene Table contains a total of 382 450 protein-coding genes from these 22 microorganisms, which have been grouped into 105 541 unique gene clusters. In the Oomycete Gene Table, the rows show gene clusters while the columns show (i) Row numbers, (ii) Cluster identification numbers (ID), (iii) Annotations based on Clusters of orthologous groups of proteins (COG) (27, 28), (iv) Functional descriptions, (v) Expected gene lengths and (vi) the organism names included in the table (Figure 1). Cluster IDs are unique identifiers that have been assigned to each of the 105 541 gene clusters (p-cluster000001-105541).

In the ‘COG annotation’ column, each code represents different COG-based protein description (e.g. COG0514 is for Superfamily II DNA helicase; COG1960 is for Acyl-CoA dehydrogenases; and COG0828 is for Ribosomal protein S21). Each COG annotation was assigned based on a BLASTP analysis of the longest protein from each gene cluster against the COG database (the BLASTP setting: sequence coverage ≥80%; protein identity ≥30%) (27, 28). The letter in square brackets indicates a COG category (e.g. [A] is for RNA processing and modification, [I] is for Lipid transport and metabolism and [K] is for Transcription). The ‘Functional description’ column displays protein functional information of the gene clusters. Each gene cluster functional description is assigned based on the most represented functional annotation among the gene members within the cluster (i.e. majority rule). Note that gene functional annotations from source databases (e.g. FungiDB, NCBI and Joint Genome Institute) are directly used without any modification for consistency purpose. The ‘Expected gene length’ column indicates the length (in base pairs) of the longest gene in each gene cluster.

Each cell in the Oomycete Gene Table provides status and data of an individual gene, such as, gene ID, length, location, CG content, annotation and sequence download links (both DNA and Protein). In each gene cluster, a light-gray cell indicates that one organism contains a sequence without a complete open reading frame that has significant sequence similarity to the genes of other organisms in the corresponding gene cluster. The lack of a complete open reading frame (or a gene) in one organism but not in the other organisms may be due to the evolutionary loss of that particular gene from the genome or the incompleteness of the analyzed genome sequence. A black cell indicates that a gene or a similar sequence is absent from an organism.

### User interface

**Figure 2:**
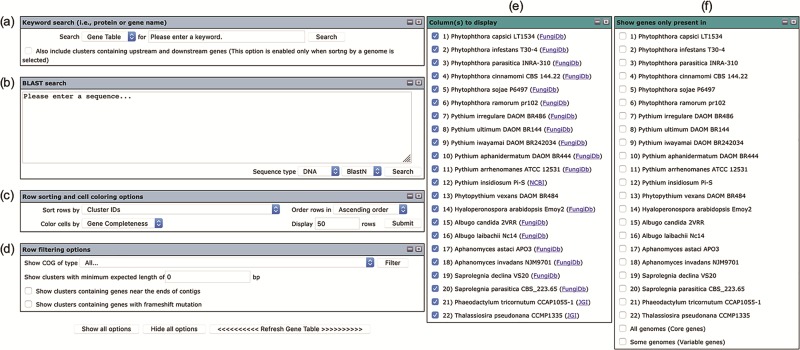
User interface of the Oomycete Gene Table comprises of six drop-down windows: (**a**) Keyword search, (**b**) BLAST search, (**c**) Row sorting and cell coloring options, (**d**) Row filtering options, (**e**) Column (s) to display and (**f**) Show genes only present in.

The user interface of the Oomycete Gene Table comprises six drop-down windows (Figure 2), which can be used to search, analyze and display the gene contents of the organisms included in the Oomycete Gene Table. The ‘Show all options’ and ‘Hide all options’ buttons can be used, respectively, to show and hide the ‘query input’ boxes and customizable options. The ‘Refresh Gene Table’ button can be used to update the data display of the Oomycete Gene Table according to the currently selected options. All display parameters can be managed and customized as described in detail below.

### Keyword Search

**Figure 3:**
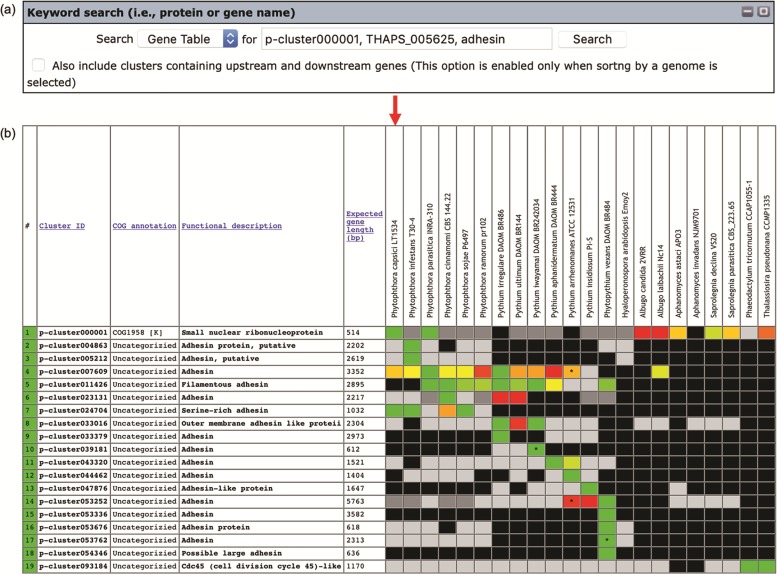
The ‘Keyword search’ window of the Oomycete Gene Table: (**a**) a single or combined keyword(s) (e.g. cluster ID, COG number and protein/gene name) can be entered in the ‘input’ box for searching the genomic data of all 22 organisms and (**b**) the Oomycete Gene Table shows a search result of the matched gene contents of all or selected genomes.

Keywords can be used to search the gene contents stored in the Oomycete Gene Table (Figure 2a). Such keywords are cluster ID (e.g. p-cluster007458 and p-cluster024940), COG IDs (e.g. COG0225, COG2183 and COG5188) or protein/gene name, protein/gene family name or protein/gene identification number (e.g. chitinase, exo1 and THAPS_005625). Multiple keywords can be used simultaneously to search the Oomycete Gene Table by separating the keywords with a comma, for example, ‘p-cluster000001, THAPS_005625, adhesin (Figure 3). Keyword search can be performed on the whole Oomycete Gene Table or focused on a specific genome by selecting from the drop-down box accordingly.

### BLAST search

For the ‘BLAST search’ window (Figure 2b), either a DNA or a protein query sequence can be entered to search for its homologous sequences in the genomes that are available in the Oomycete Gene Table. Currently, only one sequence is allowed in each search. The BLAST search result shows matched organism(s), gene/protein names, bit score(s) and *E*-value(s). Each significant match is supplemented with a link to pairwise sequence alignment of the query and subject sequences, as well as a link to view on the Oomycete Gene Table.

### Row sorting and cell coloring

**Figure 4:**
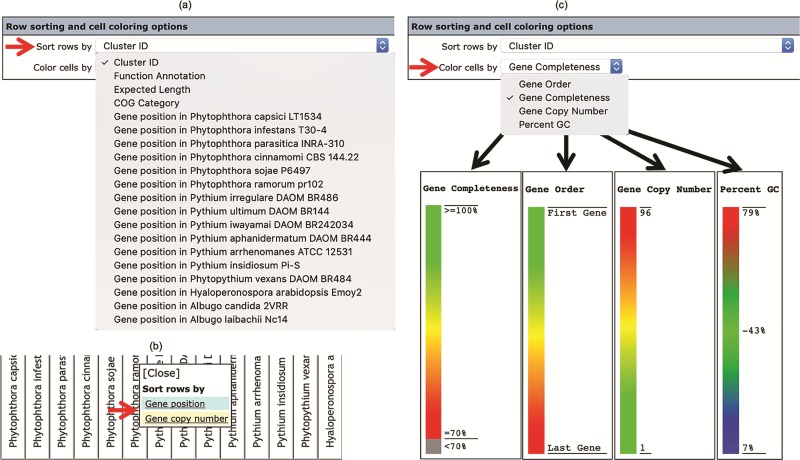
The ‘Row sorting and cell coloring’ options of the Oomycete Gene Table. Display of the gene clusters and their associated data can be customized, arranged and sorted based on: (**a**) cluster IDs, functional annotations, expected gene lengths and COG categories; (**b**) position or copy number of gene(s) in the genome; and (**c**) a selected property, such as gene completeness, gene order in a genome, gene copy number or CG content.

The number, order and cell color of the displayed gene clusters can be managed and customized using the ‘Row sorting and cell coloring’ options (Figure 2c). By default, the Oomycete Gene Table shows 50 rows of gene clusters per page in ascending order of the Cluster IDs. The cells in the displayed table are color coded to indicate the percentage of gene completeness as compared to the Expected gene length (which is the longest gene in each gene cluster). In addition to the Cluster IDs, the displayed gene clusters can be sorted according to COG annotations, functional descriptions, expected gene lengths and gene positions of a selected organism (Figure 4a). In addition, each organism name shown in the column header can be clicked to show sorting options based on gene positions or gene copy numbers in the corresponding genome (Figure 4b). The cells of the Oomycete Gene Table can be marked with different colors to demonstrate the selected property of the displayed genes (e.g. percentage of gene completeness, gene order in a genome, gene copy number or CG content) (Figure 4c).

### Row filtering

**Figure 5:**
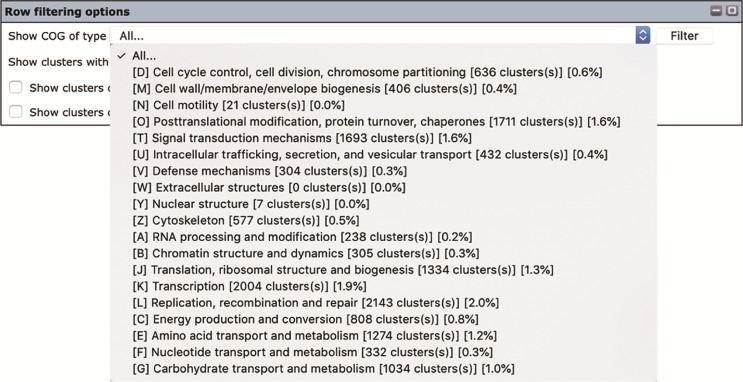
The ‘Row filtering’ options of the Oomycete Gene Table. The drop-down window is used to customize and display only gene clusters with a desired COG category. The COG codes (e.g. [D], [M] and [N]), together with the number and percentage of each gene cluster identified in the organism(s) are listed.

The ‘Row filtering’ options can be used to filter the gene contents and list only the gene clusters with a desired COG category (Figure 2d). The drop-down window shows COG functional categories (27, 28) together with numbers and percentages of gene clusters that have been assigned to them (Figure 5). A minimal length of the displayed genes can be specified by putting in a desired gene length (in base pairs) in the provided space. There are also options for displaying clusters that contain genes that are located within 300 bp from the ends of the genomic contigs (an asterisk is marked in the cell) as well as genes with possible frameshift mutation (the letter ‘F’ is marked in the cell).

### ‘Column(s) to display’ and ‘Show genes only present in’ options

The Oomycete Gene Table is set by default to display the gene contents of all organisms (see the organism columns in Figure 1). However, users can select and show only gene contents of the organism(s) of interest by checking the desired organism(s) shown in the ‘Column(s) to display’ box (Figure 2e). For example, when ‘*Phytophthora infestans*’ and ‘*Pythium ultimum*’ are checked, only gene contents of these two organisms are displayed upon refreshing the Oomycete Gene Table. Links to the source databases of the available genomes are also provided in this box.

The ‘Show genes only present in’ window (Figure 2f) can be used to display genes that are present only in the selected organism(s). For example, when ‘*Pythium insidiosum*’ is checked, a total of 998 *P. insidiosum*-specific gene clusters are shown in the Oomycete Gene Table. If multiple organisms are selected, only the gene clusters that are present in the selected organisms and absent from the other unselected organisms are displayed. For example, when ‘*Pythium insidiosum*’ and ‘*Pythium aphanidermatum*’ are checked, a total of 91 gene clusters that are specific to just these two oomycetes are shown. In addition, if the ‘All genomes (Core genes)’ box is checked, only the set of gene clusters that are present in all organisms are visualized. In contrast, if the ‘Some genomes (Variable genes)’ box is checked, only gene clusters that are present in at least one but not all of the organisms are displayed.

### Retrieval of genomic data

**Figure 6:**
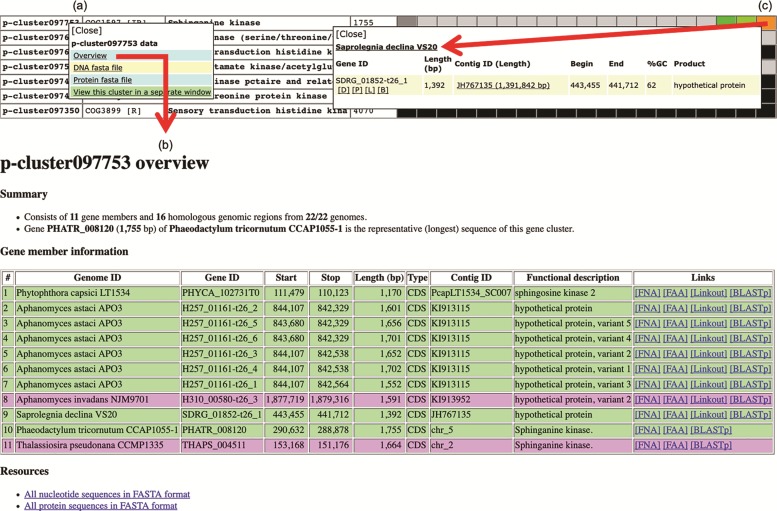
Genomic data retrieval features of the Oomycete Gene Table: (**a**) when a gene cluster ID is selected, several links are shown as gateways to access the genomic data of all organisms contained in that gene cluster; (**b**) overview genomic data includes genome IDs, contig/gene IDs, genomic location and length of the genes, functional annotations, links to gene/protein sequences, links to source databases and links to NCBI Protein-BLAST tool; and (**c**) when an individual cell is selected, the genomic data of an individual organism can be obtained.

Various genomic data can be retrieved from the Oomycete Gene Table. By clicking a cluster ID of interest (e.g. p-cluster097753; Figure 6a), a pop-up window that contains links for obtaining an overview as well as displaying sequences (in FASTA format) of all genes/proteins belonging to the corresponding gene cluster is shown. The overview page contains detail of gene members such as genome IDs, gene/contig IDs, gene locations, gene lengths, functional descriptions, links to gene/protein sequences, links to source databases and links to NCBI Protein-BLAST tool (Figure 6b). This information can also be retrieved by clicking a cell on the displayed Oomycete Gene Table. For example, Figure 6c shows the retrievable gene data of ‘*Saprolegnia declina*’ that is found in the gene cluster ‘p-cluster097753’.

## Discussion

Gene gain, loss and modification can accumulatively occur in different genomes and contribute to variations in lifestyles, pathogenicity and host specificities of the saprophytic and pathogenic oomycetes. Genome sequences of many oomycetes (6, 11–22) are now available and serve as an important resource for comparative genomic studies. Among the microbiologists and researchers in the field of oomycete molecular genetics, the lack of bioinformatic experiences and technical skills limits their capabilities to thoroughly use the available genome data to explore biology, pathogenicity and evolution of oomycetes. We have developed an online user-friendly bioinformatic tool called ‘Oomycete Gene Table’ to facilitate comparative analysis and data retrieval of oomycete genomes (3). The interface of the Oomycete Gene Table has been designed for users who are unfamiliar with or have limited experiences in big data mining and analysis of multiple genomes.

A large repertoire of ~380 000 genes from 20 oomycetes and 2 diatom genomes that have been grouped into ~100 000 homologous gene clusters can be found and analyzed in the Oomycete Gene Table. A number of useful features have been implemented, which allow users to easily search, retrieve and compare gene(s) of interest across the available genomes. Core genes of selected oomycetes can be extracted from the Oomycete Gene Table for phylogenomic analyses. Some core genes of oomycetes can be potential targets for drug design and development. Species-specific genes are important for elucidating the lifestyle and pathogenicity of a particular oomycete. For example, unlike most other pathogenic oomycetes that infect plants, *P. insidiosum* and *P. aphanidermatum* are capable of infecting humans. The Oomycete Gene Table can identify ~90 gene clusters that are only present in these two oomycetes, which may possibly contribute to their specific virulence in the human host.

Other applications of the Oomycete Gene Table include the identification of elicitins (3) and ureases (25) among oomycetes. Elicitins form a protein family that is originally identified in *Phytophthora* and *Pythium* species but not in other oomycetes or other organisms (29–31). The Oomycete Gene Table has been used to explore the presence of elicitins in various oomycetes (3). By simply using the keyword ‘elicitin’ in a search (as demonstrated in Figure 3), the result shows that *Phytophthora* and *Pythium* species contain a more extensive repertoire of the elicitin genes than the other oomycetes. Regarding the ureases, some pathogens (e.g. the bacterium *Helicobacter pylori* and the fungus *Cryptococcus neoformans*) produce urease as a virulence factor to facilitate their pathogenesis (32–34). Since the role of urease in biology and pathogenesis of oomycetes has not been characterized, the Oomycete Gene Table was searched by using the keyword ‘urease’ to identify urease-encoding genes in oomycetes (25). The proper function of urease requires the presence of several urease accessory proteins (e.g. urease accessory protein D, F and G) (35). The Oomycete Gene Table shows that although the ureases are conserved in oomycetes, the presence of urease accessory protein-encoding genes are markedly diverse in these organisms (25).

As shown above, the Oomycete Gene Table is a useful bioinformatic tool. However, it is important to note that the quality of the genome sequences included in the database, all of which are still incomplete, may compromise the reliability of analysis results. For example, an absence of a gene in one organism may actually be due to a missing or unknown genome sequence. It is also important to note that since genome data in the Oomycete Gene Table are obtained from various public resources, they may not be annotated with the same annotation pipeline. Users should always consider these limitations when interpreting the comparative analysis results. As genome sequencing technologies continue to improve in term of cost and efficiency, better quality genome sequences of oomycetes will be available and can be added into the Oomycete Gene Table to improve the database quality.

In conclusion, we described here the Oomycete Gene Table, which is an online bioinformatic tool for comparative analysis and data retrieval of oomycete genomes. The Oomycete Gene Table is accessible via a web browser, and its functionalities can be operated via an easy-to-use interface. The inexperienced users (e.g. microbiologists, researchers and clinicians) in the field can seamlessly analyze and compare the big data of multiple oomycete genomes. The genomic data in the Oomycete Gene Table will be updated regularly to increase their completeness and accuracy, which will allow the Oomycete Gene Table to serve as a useful bioinformatic tool for exploring the biology, pathogenicity and evolution of oomycetes.

## Notes

**Database URL:** http://www.sbi.kmutt.ac.th/cgi-bin/gt/viewer?organism=oomycetes&build=190208
